# Factors affecting walking ability in female patients with rheumatoid arthritis

**DOI:** 10.1371/journal.pone.0195059

**Published:** 2018-03-27

**Authors:** Yugo Morita, Hiromu Ito, Mie Torii, Akiko Hanai, Moritoshi Furu, Motomu Hashimoto, Masao Tanaka, Masayuki Azukizawa, Hidenori Arai, Tsuneyo Mimori, Shuichi Matsuda

**Affiliations:** 1 The Department of Orthopedic Surgery, Kyoto University Graduate School of Medicine, Kyoto, Japan; 2 The Department of Human Health Sciences, Kyoto University Graduate School of Medicine, Kyoto, Japan; 3 The Department of the Advanced Medicine for Rheumatic Diseases, Kyoto University Graduate School of Medicine, Kyoto, Japan; 4 National Center for Geriatrics and Gerontology, Obu, Japan; 5 The Department of Rheumatology and Clinical Immunology, Kyoto University Graduate School of Medicine, Kyoto, Japan; University of Illinois at Urbana-Champaign, UNITED STATES

## Abstract

**Objective:**

To determine the factors associated with gait parameters in female patients with rheumatoid arthritis (RA).

**Methods:**

The gait analysis was performed in a large cohort of RA patients, and three basic gait parameters (step length, cadence and gait speed) were calculated. Clinical and laboratory data were also collected. Factors associated with gait parameters were analyzed using multivariable linear regression in the three models with forced entry. Then, we divided those patients with Health Assessment Questionnaire disability index (HAQ) scores ≤ 0.5 into two groups according to their gait speed that were compared to identify the characteristics of patients with a good HAQ score but poor walking ability.

**Results:**

A total of 318 female patients were analyzed. Knee extension strength had the strongest positive association with all three gait parameters (P < 0.0001), while methotrexate use was also positively associated with all three gait parameters (step length: P < 0.05, cadence: P < 0.05 in model 1 and 2; P < 0.01 in model 3, gait speed: P < 0.01). The disease activity score was negatively associated with step length and gait speed (step length, gait speed: P < 0.01 in model 1 and 2; P < 0.05 in model 3). 26% of patients with good HAQ scores showed slow gait speed. Patients with good HAQ scores and slow gait speed had higher disease activity scores (P < 0.05) and lower knee extension strength (P < 0.0001) than those with good HAQ scores and normal gait speed.

**Conclusions:**

High knee extension strength, low disease activity and administration of methotrexate were strongly associated with good walking ability in female patients with RA. And, even if patients showed good HAQ scores, about quarter of those patients had poor walking ability, and they showed higher disease activity, lower knee extension strength, compared to the patients with normal gait speed.

## Introduction

Rheumatoid arthritis (RA) is a chronic autoimmune disorder that occurs in 0.5% to 1% of adults [1–3]. It causes synovial inflammation and articular erosion, and leads to progressive functional impairment. In recent years, advancements in disease-modifying anti-rheumatoid drugs (DMARDs) and tightly controlled treatment strategies (treat-to-target) have improved the health status of patients with RA [4]. Nevertheless, many patients still do not achieve disease remission and suffer progressive functional impairment [5]. A recent study reported that functional remission was not achieved in 36.6% of patients with RA 52 weeks after initiation of treatment [6].

Walking ability is an essential part of daily living that is often impaired in patients with RA [7]. The Health Assessment Questionnaire (HAQ) disability index, which is one of the most commonly used tools to measure disease-specific outcomes, includes an index of walking ability [8], but it is difficult to make a detailed evaluation of the characteristics of the walking ability of patients with RA using HAQ alone. Several previous studies have analyzed the gait parameters of patients with RA, including gait speed, step length and cadence, and have reported that patients with RA showed lower levels of these gait parameters compared with healthy controls [7, 9]. Some reported that gait speed showed a moderate correlation with modified HAQ [10], and others also reported that slow gait speed was a risk factor for persistent severe lower-extremity limitation, hospitalization, and mortality [11, 12]. However, there have been no studies analyzing the factors associated with walking disability in a large cohort of patients with RA.

In this study, we analyzed the gait parameters of patients in a single large RA cohort. The purposes of this study were (1) to clarify the characteristics of gait parameters in patients with RA, (2) to determine the clinical and laboratory factors associated with gait parameters in patients with RA, and (3) to identify patients with RA who showed slow gait speed despite having a good HAQ score, with the aim of implementing a strategy to maintain walking ability in patients with RA.

## Methods

### Study population

The study population included consecutive RA patients enrolled from the Kyoto University Rheumatoid Arthritis Management Alliance (KURAMA) cohort from April to December 2014. The KURAMA cohort was established in 2011 at the Center for Rheumatic Diseases at Kyoto University Hospital as a prospective diagnostic study with the aim of providing strict control of RA, and the clinical and laboratory data of the participants have been used for previous clinical investigations [13–15]. All patients were diagnosed as RA by using the American College of Rheumatology/European League against Rheumatism classification 1987 or 2010 criteria for RA. During the study period from April to December 2014, a total of 396 patients with RA were consecutively enrolled from the KURAMA cohort. Thirteen patients for whom some data were missing were excluded from the study. In the residual 383 patients, most of the patients were women (n = 318, 83.0%) and the number of male patients were small (n = 65, 17.0%). Therefore, the male patients were exclude from the study, and the data from 318 female patients were analyzed. This study was designed in accordance with the Helsinki declaration and was approved by the ethics committee of Kyoto University Graduate School and Faculty of Medicine. Written informed consent for this study was obtained from all participating patients.

### Data collection

Clinical and laboratory data included age (years), sex, body height (cm), body weight (kg), body mass index (kg/m^2^), duration of RA disease (years), Steinbrocker stage as a radiological measure of disease stage [16], swollen and tender joint counts (SJC and TJC, respectively), physician’s visual analog scale (dVAS), patient’s visual analog scale (pVAS), C-reactive protein (CRP) concentration (mg/dl), the titers of rheumatoid factor (RF, U/ml) and anti-citrullinated protein antibodies (ACPA, U/ml), HAQ score, modified Health Assessment Questionnaire disability index (mHAQ) score, and total number of total hip, knee, and ankle arthroplasties (THA, TKA, and TAA, respectively). Autoantibody titers were considered positive at > 15 U/ml for RF and at > 4.5 for ACPA. HAQ and mHAQ scores are self-reported questionnaires that assesses the physical function of patients with RA [8, 17]. Low HAQ and low mHAQ scores represent high functional status, and an HAQ score ≤ 0.5 has been defined as one criterion of minimal disease activity of RA [18, 19]. We evaluated disease activity using the Disease Activity Score 28-CRP (DAS28-CRP) and the Clinical Disease Activity Index (CDAI). Disease activity scores were reported to be positively correlated with HAQ [20]. The use of steroids, methotrexate and biological DMARDs (bDMARDs) and any comorbid interstitial lung disease were also recorded.

Knee extension strength was measured with a hand-held dynamometer (μTas F-1; ANIMA Co., Tokyo, Japan) during isometric contraction for five seconds [21, 22]. The procedure for measurement was kept consistent. The patients sat on the measurement table with their hip and knee joints at angles of 90°. The hand-held dynamometer sensor was secured to the distal front of the lower leg with a belt, and that belt was also fixed firmly to the leg of the measurement table. Measurements were taken for both legs, and the mean value of the knee extension strength was defined as the knee extension strength of the patient.

The gait was measured with a portable triaxial accelerometer rhythmogram device (Mimamori-Gait^®^ System, LSI Medience Co., Tokyo, Japan, size 8 × 6 × 2 cm, weight 80 g). Patients were asked to walk at their comfortable speed. The device was fixed firmly to the middle of the patient’s lower back and three-dimensional acceleration signals were collected every 100 Hz and automatically recorded on a secure digital memory card inserted into it for later analysis. While the patient was walking, he or she was followed by a rehabilitation professional who pushed the event marker button when the patient crossed the assessment start and finish lines. The walking data was determined from these event marker recordings so that they did not include the acceleration and deceleration walk. The number of stride events was identified from the three-dimensional acceleration signals using an automated peak-detection algorithm, and three basic gait parameters (step length, cadence, and gait speed) were calculated for each patient [23, 24]. The method we used to measure the basic gait parameters with the triaxial accelerometer was previously validated. Its inter-rater reliability and intra-rater reliability are thought to be excellent [25]. Additionally, we measured the basic gait parameters of the three healthy men using our triaxial accelerometer and a tridimensional 8-camera motion capture system (VICON^®^, VICON Motion System Ltd, Oxford, UK) [26], and compared the data between them. When we measure the basic gait parameters using VICON^®^, spherical retro-reflective markers were placed bilaterally following the Plug-in-Gait protocol, marker data were sampled at 100Hz, and the basic gait parameters were calculated using Vicon polygon 4.2 software. We evaluated the agreement between these two systems using intraclass correlation coefficient and the Pearson correlation coefficient, and found that intraclass correlation coefficient for gait speed, step length and cadence are 0.99, 0.96 and 0.99, and the Pearson correlation coefficient for gait speed, step length and cadence are 0.99, 0.97 and 0.99, respectively.

### Statistical analysis

The JMP Pro statistical package, version 11.0.0 (SAS Institute Inc., Cary, NC, USA) was used for all statistical analyses, and *P* values less than 0.05 were considered significant. Data for continuous variables were expressed as mean and standard deviation, and data for categorical variables were expressed as numbers and percentages. For between-group comparisons, we used the Wilcoxon rank-sum test for continuous variables and the chi-square test for categorical variables. The Pearson correlation coefficient (*r*) was used to assess the relationships between the three basic gait parameters (step length, cadence, and gait speed), the relationship between age and these three parameters and the relationship between these three parameters and HAQ or mHAQ. Univariate and multivariate linear regression analysis was used to identify variables that were associated with the three gait parameters (dependent variables). We constructed three models with forced entry for the multivariate linear regression analysis. In model 1, we used clinical variables as the independent variables: age, body height, body weight, duration of RA disease, Steinbrocker stage, DAS28-CRP score, steroid use, methotrexate use, bDMARDs use, comorbid interstitial lung disease, knee extension strength, and total number of THA, TKA, and THA. In model 2, we added the laboratory variables RF and ACPA as independent variables. We were unable to add CRP concentration in model 2 because the DAS-28 CRP disease activity score includes the CRP concentration and was therefore collinear with CRP concentration. In model 3, we excluded DAS-28 CRP and replaced it with the CDAI. Because the CDAI disease activity score does not include CRP concentration, we could also add CRP concentration as an independent variable in model 3. Categorical variables were coded as follows: Steinbrocker stage: 1 (stage I), 2 (stage II), 3 (stage III), or 4 (stage IV); RF: 0 (negative) or 1 (positive); ACPA: 0 (negative) or 1 (positive); steroid use: 0 (absent) or 1 (present); methotrexate use: 0 (absent) or 1 (present); bDMARDs use: 0 (absent) or 1 (present); interstitial lung disease: 0 (absent) or 1 (present). We calculated standardized partial regression coefficient (β) and *P* values for the regression analysis. In addition, we divided patients with HAQ scores ≤ 0.5 into two groups according to their gait speed, to analyze the characteristics of patients who had a good HAQ score but poor walking ability. We defined the cut-off point for gait speed as 1 m/s, because previous studies have reported that gait speed < 1 m/s is a risk factor for persistent severe lower-extremity limitation, hospitalization, and mortality [11, 12].

## Results

### Study population

A total of 318 female RA patients were analyzed. The average age at enrollment was 61.7 ± 13.4 years, and the duration of RA disease was 13.4 ± 12.5 years. Demographic data of study participants were shown in Table 1.

**Table 1:** Demographic data.

Age, years	61.7 ± 13.4
Body height, cm	155.4 ± 7.1
Body weight, kg	51.1 ± 8.4
Body mass index, kg/m^2^	21.2 ± 3.2
Disease duration of RA, years	13.4 ± 12.5
Steinbrocker Stage, no. (%)	I; 66 (20.8), II: 74 (23.3),
	III; 56 (17.6), IV; 122 (38.4)
DAS28-CRP	1.94 ± 0.84
CDAI	5.92 ± 6.28
TJC, no.	0.87 ± 1.58
SJC, no	0.83 ± 0.09
dVAS	10.2 ± 11.9
pVAS	29.7 ± 24.9
CRP, mg/dl	0.34 ± 0.04
RF positive, no. (%)	233 (73.3)
ACPA positive, no. (%)	253 (79.6)
HAQ	0.77 ± 0.76
mHAQ	0.43 ± 0.03
Steroid use, no. (%)	107 (33.65)
Methotrexate use, no. (%)	216 (67.92)
Biologics use, no (%)	133 (41.82)
Interstitial pneumonia, no. (%)	50 (15.72)
Knee extension strength, N	1678.4 ± 693.3
Total number of THA, TKA and TAA, no.	0.29 ± 0.78

Data are mean ± standard deviation or n (%). DAS28-CRP, Disease Activity Score-CRP; CDAI, Clinical Disease Activity Index; TJC, tenderness joint count; SJC, swollen joint count; dVAS, Dr's visual analog scale; pVAS, patients' visual analog scale; CRP, C-reactive protein; RF, rheumatoid factor; ACPA, anti-citrullinated protein antibodies; HAQ, Health Assessment Questionnaire disability index; mHAQ, modified Health Assessment Questionnaire disability index; THA, total hip arthroplasty; TKA, total knee arthroplasty; TAA, total ankle arthroplasty.

### Distribution of gait parameters and correlations between gait parameters and age

**Fig 1:**
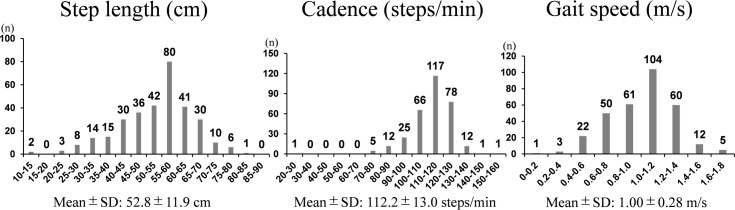
Distribution of three basic gait parameters in female patients with rheumatoid arthritis. SD, standard deviation.

**Fig 2:**
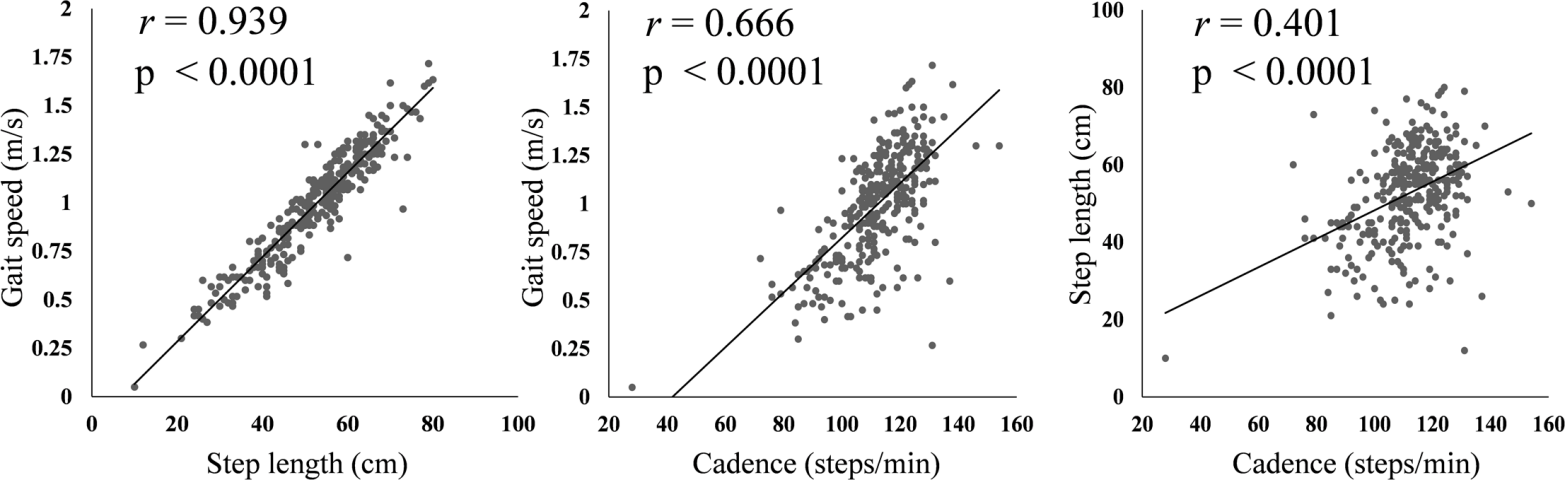
Correlations between three basic gait parameters. Correlation plot demonstrated the very strong, strong and moderate correlations between three basic gait parameters. *r* values = Pearson correlation coefficient.

**Fig 3:**
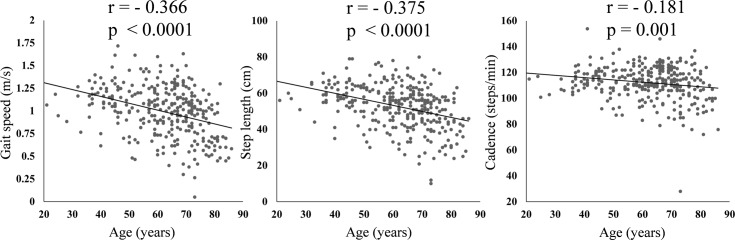
Correlations between age and three basic gait parameters. Correlation plot demonstrated the moderate and weak correlations between age and three basic gait parameters. r values = Pearson correlation coefficient.

The distribution of the three basic gait parameters in female RA patients is shown in Fig 1. Their mean step length was 52.8 ± 11.9 cm, cadence 112.2 ± 13.0 steps/min, and gait speed 1.00 ± 0.28 m/s. We next evaluated the correlations between these three basic gait parameters using the Pearson correlation coefficient. Step length was strongly correlated with gait speed (*r* = 0.939, *P* < 0.0001), as was cadence (*r* = 0.666, *P* < 0.0001). The correlation between cadence and step length was moderate (*r* = 0.401, *P* < 0.0001), and there were some RA patients who had a short step length but a fast cadence, suggesting that they compensated for their short steps with a fast cadence (Fig 2). We also evaluated the correlation between age and these three basic gait parameters. Gait speed and step length was moderately negatively correlated with age (*r* = -0.366, *P* < 0.0001 and *r* = 0.375, *P* < 0.0001, respectively). Cadence had weak negative correlation with age (*r* = - 0.181, *P* = 0.001) (Fig 3).

### Clinical and laboratory factors associated with gait parameters

We used univariate and multivariate linear regression analysis to determine which variables were associated with the three basic gait parameters of step length, cadence, and gait speed. We constructed three forced-entry models and calculated standardized partial regression coefficient (β) and *P* values. In the univariate linear regression analysis, most variables were significantly associated with one or more gait parameters (Table 2). The results of multivariate linear regression analysis using the three models were similar (S1–S3 Tables), and are summarized in Table 3. In all three models, knee extension strength had the strongest positive association with the three basic gait parameters and methotrexate use was also significantly positively associated with all three basic gait parameters. The disease activity scores (DAS-28CRP and CDAI) and the presence of comorbid interstitial lung disease were negatively associated with step length and gait speed in all three models. The total number of THA, TKA, and TAA was significantly negatively associated with step length in all three models, but showed no significant association with cadence or gait speed.

**Table 2:** Univariate linear regression analysis between gait parameters and clinical and laboratory variables.

	Step length		Cadence		Gait speed	
	β	P	β	P	β	P
Age	-0.38	<0.0001	-0.18	0.0012	-0.37	<0.0001
Body height	0.35	<0.0001	0.08	0.17	0.31	<0.0001
Body weight	0.09	0.12	0.04	0.45	0.10	0.09
Duration of RA disease	-0.27	<0.0001	-0.08	0.17	-0.26	<0.0001
Steinbrocker Stage	-0.26	<0.0001	-0.13	0.017	-0.26	<0.0001
DAS28-CRP	-0.38	<0.0001	-0.17	0.0024	-0.36	<0.0001
CDAI	-0.32	<0.0001	-0.19	0.0005	-0.32	<0.0001
CRP	-0.15	0.0098	-0.05	0.40	-0.14	0.016
RF positive	-0.08	0.18	0.01	0.81	-0.06	0.27
ACPA positive	-0.01	0.81	0.07	0.22	0.01	0.80
Steroid use	-0.23	<0.0001	-0.15	0.0093	-0.23	<0.0001
Methotrexate use	0.16	0.0055	0.16	0.0058	0.18	0.0011
bDMARDs use	0.10	0.085	-0.01	0.90	0.09	0.13
Interstitial lung disease	-0.32	<0.0001	-0.19	0.0008	-0.33	<0.0001
Knee extension strength	0.51	<0.0001	0.32	<0.0001	0.53	<0.0001
Total number of THA, TKA and TAA	-0.31	<0.0001	-0.06	0.27	-0.27	<0.0001

β values represent standardized partial regression coefficient.

**Table 3:** Summary of multivariable linear regression analysis between gait parameters and clinical and laboratory variables.

	Step length	Cadence	Gait speed
Age			(↓↓)†
Body height	↑↑		
Body weight	↓↓		↓↓
Duration of RA disease			
Steinbrocker Stage			
DAS28-CRP or CDAI	↓↓		↓↓
CRP			
RF positive			
ACPA positive			
Steroid use	(↓↓)†		(↓)†
Methotrexate use	↑	↑↑	↑↑
bDMARDs use			
Interstitial lung disease	↓↓	(↓↓)†	↓↓
Knee extension strength	↑↑↑↑	↑↑↑	↑↑↑↑↑
Total number of THA, TKA and TAA	↓↓	** **	** **

↑, 0 < β ≤ 0.1 and P < 0.05

↑↑, 0.1 < β ≤ 0.2 and P < 0.05

↑↑↑, 0.2 < β ≤ 0.3 and P < 0.05

↑↑↑↑, 0.3 < β ≤ 0.4 and P < 0.05

↑↑↑↑↑, 0.4 < β ≤ 0.5 and P < 0.05

↓, -0.1 < β < 0 and P < 0.05

↓↓, -0.2 < β ≤ -0.1 and P < 0.05 in all the three models.

† represents statistically significant only in Model 3. β represents standardized partial regression coefficient.

### Correlations between three basic gait parameters and HAQ score or mHAQ score

**Fig 4:**
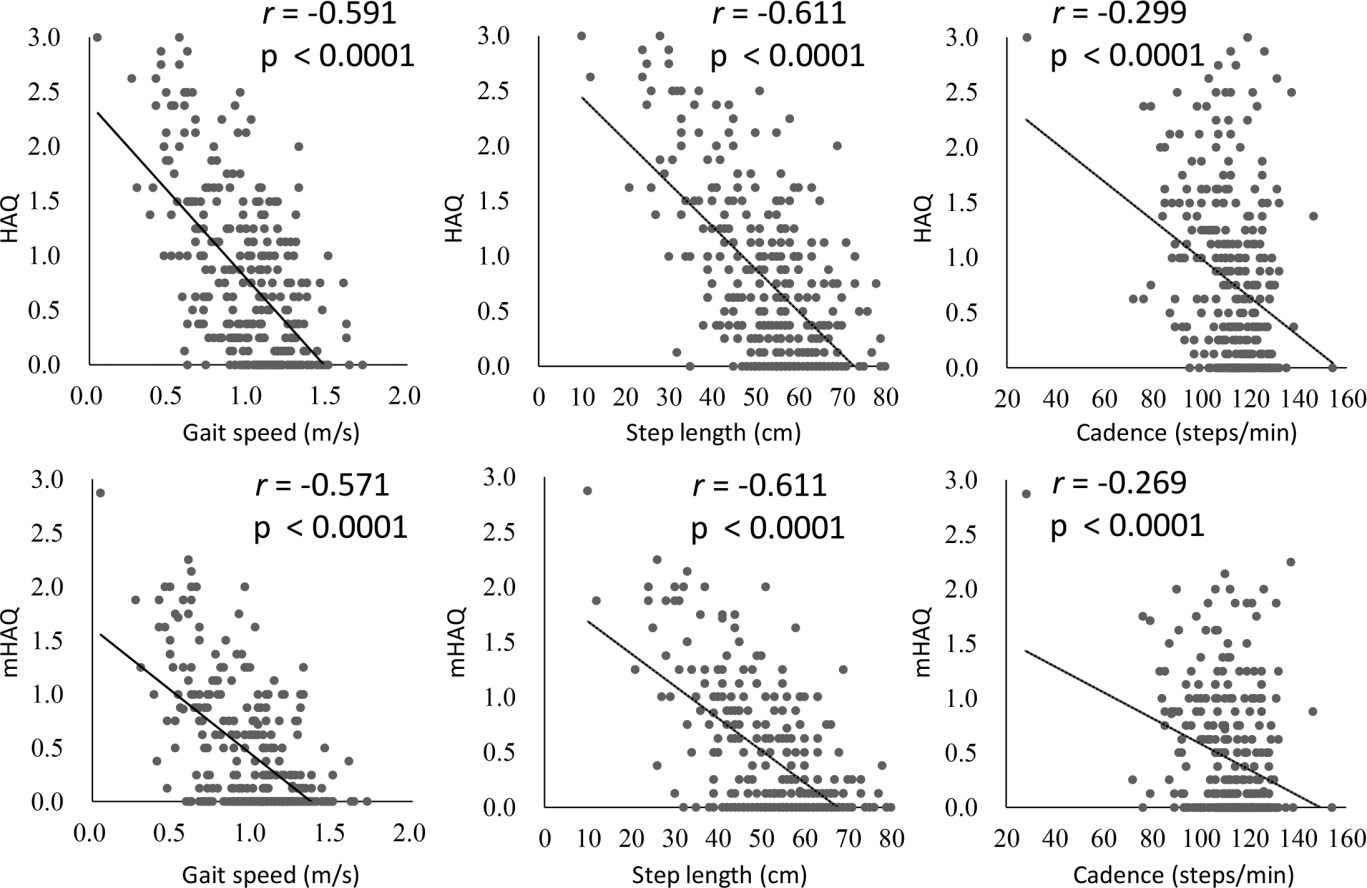
Correlation between three basic gait parameters and HAQ score or mHAQ score. Gait speed and step length had strong correlation with HAQ score and mHAQ score, but correlation between cadence and HAQ score or mHAQ score was very weak. *r* values = Pearson correlation coefficient.

We next analyzed correlation between HAQ score or mHAQ score and the three basic gait parameters to determine whether gait parameters were correlated with the physical function of patients with RA. Gait speed was strongly correlated with HAQ score and mHAQ score (*r* = –0.591, *P* < 0.0001; *r* = –0.571, *P* < 0.0001, respectively), as was step length (*r* = –0.611, *P* < 0.0001; *r* = –0.611, *P* < 0.0001, respectively). Patients with high HAQ or mHAQ scores showed low gait speed and short step length. However, cadence was only very weakly correlated with HAQ score and mHAQ score (*r* = –0.299, *P* < 0.0001; *r* = –0.269, *P* < 0.0001, respectively), and we observed that a considerable number of patients had relatively high HAQ or mHAQ scores but a relatively fast cadence (Fig 4).

### Characteristics of patients with good HAQ scores but low gait speed

HAQ is a self-reported questionnaire that assesses the physical function of patients with RA. A low HAQ score represents high functional status, and an HAQ score ≤ 0.5 has been defined as one criterion of minimal disease activity of RA [18, 19]. In this study, gait speed was strongly negatively correlated with HAQ score, but there were some patients with a low (good) HAQ score who had low gait speed (Fig 3). We divided those patients with an HAQ score ≤ 0.5 into two groups according to their gait speed, then analyzed the characteristics of patients with HAQ scores ≤ 0.5 but with low gait speed. Previous studies had reported that a gait speed < 1 m/s was a risk factor for persistent severe lower-extremity limitation, hospitalization and mortality [11, 12], so we defined this as the cut-off point between the two groups. Of the patients with HAQ scores ≤ 0.5, 114 (74.0%) had normal gait speed but 40 (26.0%) had low gait speed. Table 4 shows the differences between these two groups. Patients with a good HAQ score but low gait speed were significantly older and had significantly lower body height, longer duration of RA disease, higher disease activity score (DAS28-CRP and CDAI), higher TJC, higher pVAS, higher CRP, higher rate of interstitial lung disease and lower knee extension strength than patients with a good HAQ score and normal gait speed.

**Table 4:** Differences between patients who had slow gait speed with HAQ score ≤ 0.5 and patients who had normal gait speed with HAQ score ≤ 0.5.

	Gait speed < 1	Gait speed ≥ 1					
	(n = 40, 26.0%)	(n = 114, 74.0%)	P
Step length, cm	48.6 ± 5.7	61.3 ± 6.9	<0.0001
Cadence, steps/min	106.4 ± 8.7	118.4 ± 7.9	<0.0001
Gait speed, m/s	0.86 ± 0.12	1.21 ± 0.15	<0.0001
Age, years	62.2 ± 11.2	55.1 ± 12.9	0.0032
Body height, cm	155.5 ± 6.5	158.1 ± 6.7	0.041
Body weight, kg	53.4 ± 10.3	53.2 ± 6.8	0.06
Body mass index, kg/m^2^	21.2 ± 3.6	21.4 ± 3.0	0.62
Duration of RA disease, years	11.1 ± 9.3	7.9 ± 9.9	0.0093
Steinbrocker Stage, no.	I; 8, II; 11,	I; 44, II; 33,	0.099
	III; 9, IV; 12	III; 17, IV; 20	
DAS28-CRP	1.66 ± 0.63	1.42 ± 0.56	0.0069
CDAI	3.72 ± 3.34	2.55 ± 2.96	0.011
TJC, no.	0.63 ± 1.08	0.28 ± 0.68	0.047
SJC, no	0.50 ± 0.91	0.41 ± 0.92	0.35
dVAS	6.8 ± 7.9	5.1 ± 6.6	0.51
pVAS	17.2 ± 17.4	13.3 ± 16.8	0.042
CRP, mg/dl	0.34 ± 0.58	0.19 ± 0.58	0.045
RF positive, no. (%)	28 (70.0)	79 (69.3)	0.93
ACPA positive, no. (%)	27 (67.5)	92 (80.7)	0.087
Steroid use, no. (%)	13 (32.5)	27 (23.7)	0.27
Methotrexate use, no. (%)	30 (75.0)	84 (73.7)	0.87
bDMARDs use, no (%)	15 (37.5)	51 (44.7)	0.43
Interstitial lung disease, no. (%)	7 (17.5)	6 (5.3)	0.017
Knee extension strength, N	1478.1 ± 519.5	2109.9 ± 636.0	<0.0001
Total number of THA, TKA and TAA, no.	0.05 ± 0.22	0.06 ± 0.31	0.90

Data are mean ± standard deviation or n (%). P value were derived from Wilcoxon rank-sum test or chi-square test.

## Discussion

In the present study, we first identified correlations between three gait parameters in the female patients with RA. Step length was very strongly and cadence was strongly correlated with gait speed. However, the correlation between cadence and step length was moderate, which suggested that there were some patients who compensated for short step length by a fast cadence. We also evaluated the correlation between age and these three basic gait parameters. All the three gait parameters in female RA patients were negatively correlated with age, in spite of the fact that cadence in healthy people was reported not to decrease as they became older [27]. Second, we analyzed the clinical and laboratory factors that were associated with the three basic gait parameters. Knee extension strength had the strongest positive association with all three basic gait parameters, and methotrexate use was also positively associated with all three gait parameters. The disease activity scores (DAS28-CRP and CDAI) and the presence of interstitial lung disease were negatively associated with step length and gait speed, and the total number of THA, TKA, TAA was negatively associated only with step length. Fourth, we showed that there were some patients who had high-risk low gait speed despite having a good HAQ score. These patients typically were significantly older and had higher disease activity scores (DAS28-CRP and CDAI) and significantly lower knee extension strength than patients with a normal HAQ score and good gait speed. Even if a patient has a good HAQ score, it would be advisable to evaluate their gait parameters if they meet these criteria. To the best of our knowledge, this is the first study that has analyzed the characteristics of gait parameters in a large cohort of patients with RA and the clinical and laboratory factors associated with their walking ability.

For the female RA patients included in this study, the mean step length was 52.8 ± 11.9 cm, mean cadence was 112.2 ± 13.0 steps/min, and mean gait speed was 1.00 ± 0.28 m/s. A previous study that analyzed the results of a 10-m walking test in 119 Japanese healthy female volunteers who were 64.43 ± 10.80 years old reported that the mean step length was 67.04 ± 6.18 cm, mean cadence was 122.35 ± 7.99 steps/min, and mean gait speed was 1.37 ± 0.16 m/s [28]. Compared with these data from healthy volunteers, the step length, cadence, and gait speed of the patients in our study were all very low, which is consistent with the findings of previous studies [7, 9]. Furthermore, all three gait parameters in female RA patients had negative correlation with age in this study, in spite of the fact that cadence in healthy people was reported not to decrease as they became older [27]. These results unfortunately demonstrate that even those patients who receive currently available advanced treatments still show much poorer walking ability compared with the general population, and that their walking ability gets poorer as they become older. This should be borne in mind by all medical practitioners.

Knee extension strength provides functional stability to the knee joint. We previously reported that knee extension strength contributed to patients’ satisfaction, symptoms, and functional activities following TKA [29], and others have shown that quadriceps-specific exercise effectively improved physical function in patients with osteoarthritis and RA [30, 31]. The results of the multivariable regression analysis in this study clearly show that knee extension strength have the strongest association with gait parameters in RA patients, which may prompt physicians and societies to plan a rehabilitation program or daily exercise program to improve knee extension strength in RA patients.

DAS-28-CRP and CDAI are objective and standardized tools to evaluate disease activity and the response to treatment in patients with RA [32, 33]. These disease activity indices have been reported previously to be good indicators of functional capacity in patients with RA [20, 34]. In this study, DAS28-CRP and CDAI scores were negatively associated with step length and gait speed. Therefore, this study may also support the prevailing concept that maintaining low disease activity is crucial to sustaining good functional ability in RA patients, although this study particularly emphasized the maintenance of their walking ability.

Surprisingly, this study also showed methotrexate use was significantly positively associated with all three basic gait parameters, independent of disease activity. To clarify the reasons for this result, we evaluated the differences between patients treated and not treated with methotrexate (S4 Table). The DAS28-CRP and CDAI disease activity scores were similar in the two groups, but pVAS was significantly lower in patients treated with methotrexate than those without methotrexate. This result may suggest the possibility that methotrexate benefits patients by reducing their pain, which could contribute to maintaining their walking ability. Indeed, previous studies reported that methotrexate reduced pain not only in patients with RA but also in patients with osteoarthritis [35, 36]. However, there were difference in age between these two groups, and this study is just a cross-sectional study. This hypothesis requires testing in a longitudinal prospective study.

This study also clearly demonstrated that a part of patients with RA report good functional ability in terms of a low HAQ score but have poor walking ability. This subset of patients requires careful attention from physicians and societies. The risk factors of these patients are similar to the general risk factors associated with poorer walking ability, such as weaker knee extension strength and higher disease activity, but also suggest that older patients with longer duration of RA disease, higher pVAS, higher CRP, more TJC, and with interstitial lung disease should be monitored carefully in terms of their walking ability. These results are also consistent with those of a previous study that reported that patients with interstitial pneumonia exhibited reduced levels of physical activity in daily life and a 65% decrease in the number of steps taken per day compared with the sedentary healthy control population [37].

This study has several limitations. First, because this study was cross-sectional, it is possible that a longitudinal study would identify additional variables associated with gait parameters, and we couldn’t determine the causal relation between walking ability and the other variables. Second, we analyzed only the women because the majority of our study participants were women. Analyses of male patients might show different results. Third, we measured the knee extension strength using the hand-held dynamometer with a restraining belt. This method was previously validated, and its interrater reliability was regarded as excellent (interclass correlation coefficient: 0.98) [38]. However, the validity of this method has not been compared with the Biodex system (Biodex Medical Systems Inc., NY, USA) which is the gold standard for isometric strength testing [39]. Lastly, we hypothesized that the presence of comorbid interstitial lung disease affected the walking ability of patients with RA, which was partly validated. However, other comorbidities such as heart diseases could also affect walking ability. These factors should be investigated in future studies.

In conclusion, we analyzed the gait parameters of a cohort of patients with RA and the factors associated with those gait parameters. Knee extension strength showed the strongest positive association with three basic gait parameters, while methotrexate use was also positively associated with these three parameters. The DAS-28-CRP and CDAI disease activity scores were negatively associated with step length and gait speed. In addition, patients who reported good functional ability in terms of low HAQ scores but had poor walking ability had significantly higher disease activity scores and lower knee extension strength compared with patients who reported good functional ability and had normal walking ability. These results may suggest that it is important for walking ability in RA patients that they maintain good knee extension strength and low disease RA activity with methotrexate treatment. A further longitudinal prospective study is required.

## Supporting information

S1 Table Multivariate linear regression analysis between step length and clinical and laboratory variables. β values represent standardized partial regression coefficient. R^2^ for model 1, model 2 and model 3 are 0.430, 0.433 and 0.426, respectively. P values calculated by ANOVA were < 0.0001 in all the three models.
(DOCX)

S2 Table Multivariate linear regression analysis between cadence and clinical and laboratory variables. β values represent standardized partial regression coefficient. R^2^ for model 1, model 2 and model 3 are 0.165, 0.171 and 0.181, respectively. P values calculated by ANOVA were < 0.0001 in all the three models.
(DOCX)

S3 Table Multivariate linear regression analysis between gait speed and clinical and laboratory variables. β values represent standardized partial regression coefficient. R^2^ for model 1, model 2 and model 3 are 0.432, 0.433 and 0.434, respectively. P values calculated by ANOVA were < 0.0001 in all the three models.
(DOCX)

S4 Table Differences between patients with methotrexate use and patients without methotrexate use. Data are mean ± standard deviation or n (%). P value were derived from Wilcoxon rank-sum test or chi-square test.
(DOCX)

## References

[1] Carmona L, Villaverde V, Hernandez-Garcia C, Ballina J, Gabriel R, Laffon A. The prevalence of rheumatoid arthritis in the general population of Spain. Rheumatology (Oxford). 2002;41(1):88–95. Epub 2002/01/17. . 11792885 10.1093/rheumatology/41.1.88

[2] Gabriel SE, Crowson CS, O'Fallon WM. The epidemiology of rheumatoid arthritis in Rochester, Minnesota, 1955–1985. Arthritis Rheum. 1999;42(3):415–20. Epub 1999/03/24. doi: 10.1002/1529-0131(199904)42:3<415::AID-ANR4>3.0.CO;2-Z . 10088762 10.1002/1529-0131(199904)42:3<415::AID-ANR4>3.0.CO;2-Z

[3] Silman AJ, Pearson JE. Epidemiology and genetics of rheumatoid arthritis. Arthritis research. 2002;4 Suppl 3:S265–72. Epub 2002/07/12. doi: 10.1186/ar578 ; PubMed Central PMCID: PMCPMC3240153. 12110146 10.1186/ar578 PMC3240153

[4] Uhlig T, Heiberg T, Mowinckel P, Kvien TK. Rheumatoid arthritis is milder in the new millennium: health status in patients with rheumatoid arthritis 1994–2004. Ann Rheum Dis. 2008;67(12):1710–5. doi: 10.1136/ard.2007.084673 . 18218667 10.1136/ard.2007.084673

[5] Rubbert-Roth A, Finckh A. Treatment options in patients with rheumatoid arthritis failing initial TNF inhibitor therapy: a critical review. Arthritis Res Ther. 2009;11 Suppl 1:S1 Epub 2009/04/25. doi: 10.1186/ar2666 ; PubMed Central PMCID: PMCPMC2669237. 19368701 10.1186/ar2666 PMC2669237

[6] Sugihara T, Ishizaki T, Hosoya T, Iga S, Yokoyama W, Hirano F, et al Structural and functional outcomes of a therapeutic strategy targeting low disease activity in patients with elderly-onset rheumatoid arthritis: a prospective cohort study (CRANE). Rheumatology (Oxford). 2015;54(5):798–807. doi: 10.1093/rheumatology/keu395 . 25296748 10.1093/rheumatology/keu395

[7] Weiss RJ, Wretenberg P, Stark A, Palmblad K, Larsson P, Grondal L, et al Gait pattern in rheumatoid arthritis. Gait Posture. 2008;28(2):229–34. doi: 10.1016/j.gaitpost.2007.12.001 . 18226528 10.1016/j.gaitpost.2007.12.001

[8] Fries JF, Spitz P, Kraines RG, Holman HR. Measurement of patient outcome in arthritis. Arthritis Rheum. 1980;23(2):137–45. Epub 1980/02/01. . 7362664 10.1002/art.1780230202

[9] Turner DE, Helliwell PS, Siegel KL, Woodburn J. Biomechanics of the foot in rheumatoid arthritis: identifying abnormal function and the factors associated with localised disease 'impact'. Clin Biomech (Bristol, Avon). 2008;23(1):93–100. doi: 10.1016/j.clinbiomech.2007.08.009 . 17904711 10.1016/j.clinbiomech.2007.08.009

[10] Yamada M, Aoyama T, Mori S, Nishiguchi S, Okamoto K, Ito T, et al Objective assessment of abnormal gait in patients with rheumatoid arthritis using a smartphone. Rheumatol Int. 2012;32(12):3869–74. doi: 10.1007/s00296-011-2283-2 . 22193221 10.1007/s00296-011-2283-2

[11] Cesari M, Kritchevsky SB, Penninx BW, Nicklas BJ, Simonsick EM, Newman AB, et al Prognostic value of usual gait speed in well-functioning older people—results from the Health, Aging and Body Composition Study. J Am Geriatr Soc. 2005;53(10):1675–80. doi: 10.1111/j.1532-5415.2005.53501.x . 16181165 10.1111/j.1532-5415.2005.53501.x

[12] Rosano C, Newman AB, Katz R, Hirsch CH, Kuller LH. Association between lower digit symbol substitution test score and slower gait and greater risk of mortality and of developing incident disability in well-functioning older adults. J Am Geriatr Soc. 2008;56(9):1618–25. doi: 10.1111/j.1532-5415.2008.01856.x ; PubMed Central PMCID: PMCPMC2631090. 18691275 10.1111/j.1532-5415.2008.01856.x PMC2631090

[13] Iwata T, Ito H, Furu M, Hashimoto M, Fujii T, Ishikawa M, et al Periarticular osteoporosis of the forearm correlated with joint destruction and functional impairment in patients with rheumatoid arthritis. Osteoporos Int. 2016;27(2):691–701. Epub 2015/08/06. doi: 10.1007/s00198-015-3256-1 . 26243360 10.1007/s00198-015-3256-1

[14] Ishikawa M, Ito H, Furu M, Hashimoto M, Fujii T, Okahata A, et al Plasma sLOX-1 is a potent biomarker of clinical remission and disease activity in patients with seropositive RA. Mod Rheumatol. 2016;26(5):696–701. doi: 10.3109/14397595.2015.1128871 . 26911839 10.3109/14397595.2015.1128871

[15] Hamamoto Y, Ito H, Furu M, Hashimoto M, Fujii T, Ishikawa M, et al Serological and Progression Differences of Joint Destruction in the Wrist and the Feet in Rheumatoid Arthritis—A Cross-Sectional Cohort Study. PLoS One. 2015;10(8):e0136611 doi: 10.1371/journal.pone.0136611 ; PubMed Central PMCID: PMCPMC4552680. 26317770 10.1371/journal.pone.0136611 PMC4552680

[16] Steinbrocker O, Traeger CH, Batterman RC. Therapeutic criteria in rheumatoid arthritis. Journal of the American Medical Association. 1949;140(8):659–62. Epub 1949/06/25. . 18150288 10.1001/jama.1949.02900430001001

[17] Pincus T, Summey JA, Soraci SA Jr., Wallston KA, Hummon NP. Assessment of patient satisfaction in activities of daily living using a modified Stanford Health Assessment Questionnaire. Arthritis Rheum. 1983;26(11):1346–53. Epub 1983/11/01. . 6639693 10.1002/art.1780261107

[18] Wells GA, Boers M, Shea B, Brooks PM, Simon LS, Strand CV, et al Minimal disease activity for rheumatoid arthritis: a preliminary definition. J Rheumatol. 2005;32(10):2016–24. Epub 2005/10/06. . 16206362

[19] Pietrapertosa D, Salaffi F, Peluso G, Bosello SL, Fedele AL, Cuoghi I, et al Residual minimal disease activity in rheumatoid arthritis: a simple definition through an in-depth statistical analysis of the major outcome. Rheumatology (Oxford). 2009;48(10):1242–6. doi: 10.1093/rheumatology/kep217 . 19635732 10.1093/rheumatology/kep217

[20] Aletaha D, Nell VP, Stamm T, Uffmann M, Pflugbeil S, Machold K, et al Acute phase reactants add little to composite disease activity indices for rheumatoid arthritis: validation of a clinical activity score. Arthritis Res Ther. 2005;7(4):R796–806. Epub 2005/07/01. doi: 10.1186/ar1740 ; PubMed Central PMCID: PMCPMC1175030. 15987481 10.1186/ar1740 PMC1175030

[21] Nomura T, Ishiguro T, Ohira M, Ikeda Y. Regular exercise behavior is related to lower extremity muscle strength in patients with type 2 diabetes: Data from the Multicenter Survey of the Isometric Lower Extremity Strength in Type 2 Diabetes study. J Diabetes Investig. 2017 doi: 10.1111/jdi.12703 . 28613394 10.1111/jdi.12703 PMC5835449

[22] Amano T, Tamari K, Tanaka S, Uchida S, Ito H, Morikawa S, et al Factors for Assessing the Effectiveness of Early Rehabilitation after Minimally Invasive Total Knee Arthroplasty: A Prospective Cohort Study. PLoS One. 2016;11(7):e0159172 doi: 10.1371/journal.pone.0159172 ; PubMed Central PMCID: PMCPMC4943652. 27410385 10.1371/journal.pone.0159172 PMC4943652

[23] Yoneyama M, Kurihara Y, Watanabe K, Mitoma H. Accelerometry-based gait analysis and its application to Parkinson's disease assessment—part 1: detection of stride event. IEEE Trans Neural Syst Rehabil Eng. 2014;22(3):613–22. doi: 10.1109/TNSRE.2013.2260561 . 23661322 10.1109/TNSRE.2013.2260561

[24] Hatanaka N, Sato K, Hishikawa N, Takemoto M, Ohta Y, Yamashita T, et al Comparative Gait Analysis in Progressive Supranuclear Palsy and Parkinson's Disease. Eur Neurol. 2016;75(5–6):282–9. doi: 10.1159/000445111 . 27288001 10.1159/000445111

[25] Miyata R, Matsumoto S, Miura S, Kawamura K, Uema T, Miyara K, et al Intra-rater and inter-rater reliability of the portable gait rhythmogram in post-stroke patients. Journal of physical therapy science. 2017;29(5):874–9. Epub 2017/06/13. doi: 10.1589/jpts.29.874 ; PubMed Central PMCID: PMCPMC5462690. 28603363 10.1589/jpts.29.874 PMC5462690

[26] Daniilidis K, Jakubowitz E, Thomann A, Ettinger S, Stukenborg-Colsman C, Yao D. Does a foot-drop implant improve kinetic and kinematic parameters in the foot and ankle? Archives of orthopaedic and trauma surgery. 2017;137(4):499–506. Epub 2017/02/22. doi: 10.1007/s00402-017-2652-8 . 28220261 10.1007/s00402-017-2652-8

[27] Samson MM, Crowe A, de Vreede PL, Dessens JA, Duursma SA, Verhaar HJ. Differences in gait parameters at a preferred walking speed in healthy subjects due to age, height and body weight. Aging (Milan, Italy). 2001;13(1):16–21. Epub 2001/04/09. . 11292147 10.1007/BF03351489

[28] Yoneyama M, Mitoma H, Hayashi A. EFFECT OF AGE, GENDER, AND WALKWAY LENGTH ON ACCELEROMETRY-BASED GAIT PARAMETERS FOR HEALTHY ADULT SUBJECTS. Journal of Mechanics in Medicine and Biology. 2016;16(3). doi: 10.1142/s0219519416500299 PubMed PMID: WOS:000376382100011.

[29] Furu M, Ito H, Nishikawa T, Nankaku M, Kuriyama S, Ishikawa M, et al Quadriceps strength affects patient satisfaction after total knee arthroplasty. J Orthop Sci. 2016;21(1):38–43. doi: 10.1016/j.jos.2015.10.002 . 26755384 10.1016/j.jos.2015.10.002

[30] Juhl C, Christensen R, Roos EM, Zhang W, Lund H. Impact of exercise type and dose on pain and disability in knee osteoarthritis: a systematic review and meta-regression analysis of randomized controlled trials. Arthritis Rheumatol. 2014;66(3):622–36. doi: 10.1002/art.38290 . 24574223 10.1002/art.38290

[31] McMeeken J, Stillman B, Story I, Kent P, Smith J. The effects of knee extensor and flexor muscle training on the timed-up-and-go test in individuals with rheumatoid arthritis. Physiotherapy research international: the journal for researchers and clinicians in physical therapy. 1999;4(1):55–67. Epub 1999/06/16. . 10368839 10.1002/pri.1999.4.1.55

[32] Inoue E, Yamanaka H, Hara M, Tomatsu T, Kamatani N. Comparison of Disease Activity Score (DAS)28- erythrocyte sedimentation rate and DAS28- C-reactive protein threshold values. Ann Rheum Dis. 2007;66(3):407–9. doi: 10.1136/ard.2006.054205 ; PubMed Central PMCID: PMCPMC1856019. 16926186 10.1136/ard.2006.054205 PMC1856019

[33] Gaujoux-Viala C, Mouterde G, Baillet A, Claudepierre P, Fautrel B, Le Loet X, et al Evaluating disease activity in rheumatoid arthritis: which composite index is best? A systematic literature analysis of studies comparing the psychometric properties of the DAS, DAS28, SDAI and CDAI. Joint Bone Spine. 2012;79(2):149–55. doi: 10.1016/j.jbspin.2011.04.008 . 21680221 10.1016/j.jbspin.2011.04.008

[34] Welsing PM, van Gestel AM, Swinkels HL, Kiemeney LA, van Riel PL. The relationship between disease activity, joint destruction, and functional capacity over the course of rheumatoid arthritis. Arthritis Rheum. 2001;44(9):2009–17. Epub 2001/10/11. doi: 10.1002/1529-0131(200109)44:9<2009::AID-ART349>3.0.CO;2-L . 11592361 10.1002/1529-0131(200109)44:9<2009::AID-ART349>3.0.CO;2-L

[35] Kremer JM, Phelps CT. Long-term prospective study of the use of methotrexate in the treatment of rheumatoid arthritis. Update after a mean of 90 months. Arthritis Rheum. 1992;35(2):138–45. Epub 1992/02/01. . 1734902 10.1002/art.1780350203

[36] Wenham CY, Grainger AJ, Hensor EM, Caperon AR, Ash ZR, Conaghan PG. Methotrexate for pain relief in knee osteoarthritis: an open-label study. Rheumatology (Oxford). 2013;52(5):888–92. doi: 10.1093/rheumatology/kes386 . 23300331 10.1093/rheumatology/kes386

[37] Wallaert B, Monge E, Le Rouzic O, Wemeau-Stervinou L, Salleron J, Grosbois JM. Physical activity in daily life of patients with fibrotic idiopathic interstitial pneumonia. Chest. 2013;144(5):1652–8. doi: 10.1378/chest.13-0806 . 23928896 10.1378/chest.13-0806

[38] Katoh M, Yamasaki H. Comparison of Reliability of Isometric Leg Muscle Strength Measurements Made Using a Hand-Held Dynamometer with and without a Restraining Belt. Journal of physical therapy science. 2009;21(1):37–42. doi: 10.1589/jpts.21.37 PubMed PMID: WOS:000264575600006.

[39] Ha M, Han D. The relationship between knee joint angle and knee flexor and extensor muscle strength. Journal of physical therapy science. 2017;29(4):662–4. Epub 2017/05/24. doi: 10.1589/jpts.29.662 ; PubMed Central PMCID: PMCPMC5430269. 28533606 10.1589/jpts.29.662 PMC5430269

