# Supplementary material for: Factors affecting walking ability in female patients with rheumatoid arthritis
Source: PLoS One. 2018 Mar 27;13(3):e0195059. doi: 10.1371/journal.pone.0195059 (PMC5870996; doi:10.1371/journal.pone.0195059)
Supplement: S1 Table — β values represent standardized partial regression coefficient. R2 for model 1, model 2 and model 3 are 0.430, 0.433 and 0.426, respectively. P values calculated by ANOVA were < 0.0001 in all the three models. (DOCX) [file pone.0195059.s001.docx]

| **S1 Table. Multivariate linear regression analysis between step length and clinical and laboratory variables.** | | | | | | |
| --- | --- | --- | --- | --- | --- | --- |
|  | Model 1 | | Model 2 | | Model 3 | |
|  | β | P | β | P | β | P |
| Age | −0.09 | 0.083 | −0.10 | 0.073 | −0.11 | 0.042 |
| Body height | 0.14 | 0.0098 | 0.14 | 0.011 | 0.13 | 0.019 |
| Body weight | −0.14 | 0.0041 | −0.14 | 0.0045 | −0.14 | 0.0036 |
| Duration of RA disease | −0.05 | 0.38 | −0.05 | 0.39 | −0.03 | 0.61 |
| Steinbrocker Stage | 0.01 | 0.95 | 0.01 | 0.84 | −0.02 | 0.73 |
| DAS28-CRP | −0.14 | 0.0045 | −0.14 | 0.0068 |  |  |
| CDAI |  |  |  |  | −0.10 | 0.040 |
| CRP |  |  |  |  | 0.05 | 0.35 |
| RF positive |  |  | −0.04 | 0.46 | −0.06 | 0.27 |
| ACPA positive |  |  | −0.01 | 0.97 | 0.01 | 0.96 |
| Steroid use | −0.08 | 0.10 | −0.08 | 0.090 | −0.10 | 0.041 |
| Methotrexate use | 0.09 | 0.040 | 0.09 | 0.047 | 0.10 | 0.030 |
| bDMARDs use | 0.05 | 0.31 | 0.04 | 0.34 | 0.04 | 0.33 |
| Interstitial lung disease | −0.13 | 0.0053 | −0.13 | 0.0059 | −0.14 | 0.0044 |
| Knee extension strength | 0.36 | <0.0001 | 0.37 | <0.0001 | 0.37 | <0.0001 |
| Total number of THA, TKA and TAA | −0.10 | 0.038 | −0.10 | 0.034 | −0.11 | 0.022 |
| β values represent standardized partial regression coefficient. R^2^ for model 1, model 2 and model 3 are 0.430, 0.433 and 0.426, respectively. P values calculated by ANOVA were < 0.0001 in all the three models. | | | | | | |
