# Supplementary material for: Factors affecting walking ability in female patients with rheumatoid arthritis
Source: PLoS One. 2018 Mar 27;13(3):e0195059. doi: 10.1371/journal.pone.0195059 (PMC5870996; doi:10.1371/journal.pone.0195059)
Supplement: S2 Table — β values represent standardized partial regression coefficient. R2 for model 1, model 2 and model 3 are 0.165, 0.171 and 0.181, respectively. P values calculated by ANOVA were < 0.0001 in all the three models. (DOCX) [file pone.0195059.s002.docx]

| **S2 Table. Multivariate linear regression analysis between cadence and clinical and laboratory variables.** | | | | | | |
| --- | --- | --- | --- | --- | --- | --- |
|  | Model 1 | | Model 2 | | Model 3 | |
|  | β | P | β | P | β | P |
| Age | −0.09 | 0.17 | −0.08 | 0.21 | −0.09 | 0.15 |
| Body height | −0.09 | 0.16 | −0.09 | 0.15 | −0.11 | 0.090 |
| Body weight | −0.05 | 0.42 | −0.04 | 0.48 | −0.05 | 0.41 |
| Duration of RA disease | 0.06 | 0.43 | 0.06 | 0.38 | 0.08 | 0.23 |
| Steinbrocker Stage | −0.07 | 0.34 | −0.09 | 0.23 | −0.12 | 0.11 |
| DAS28-CRP | −0.08 | 0.17 | −0.09 | 0.15 |  |  |
| CDAI |  |  |  |  | −0.11 | 0.057 |
| CRP |  |  |  |  | 0.10 | 0.083 |
| RF positive |  |  | 0.00 | 0.99 | −0.02 | 0.73 |
| ACPA positive |  |  | 0.08 | 0.16 | 0.09 | 0.13 |
| Steroid use | −0.04 | 0.44 | −0.04 | 0.45 | −0.06 | 0.28 |
| Methotrexate use | 0.13 | 0.016 | 0.13 | 0.015 | 0.14 | 0.0083 |
| bDMARDs use | −0.03 | 0.60 | −0.03 | 0.59 | −0.03 | 0.62 |
| Interstitial lung disease | −0.10 | 0.076 | −0.11 | 0.064 | −0.12 | 0.037 |
| Knee extension strength | 0.29 | <0.0001 | 0.29 | <0.0001 | 0.29 | <0.0001 |
| Total number of THA, TKA and TAA | 0.05 | 0.38 | 0.05 | 0.35 | 0.05 | 0.37 |
| β values represent standardized partial regression coefficient. R^2^ for model 1, model 2 and model 3 are 0.165, 0.171 and 0.181, respectively. P values calculated by ANOVA were < 0.0001 in all the three models. | | | | | | |
