# Supplementary material for: Factors affecting walking ability in female patients with rheumatoid arthritis
Source: PLoS One. 2018 Mar 27;13(3):e0195059. doi: 10.1371/journal.pone.0195059 (PMC5870996; doi:10.1371/journal.pone.0195059)
Supplement: S3 Table — β values represent standardized partial regression coefficient. R2 for model 1, model 2 and model 3 are 0.432, 0.433 and 0.434, respectively. P values calculated by ANOVA were < 0.0001 in all the three models. (DOCX) [file pone.0195059.s003.docx]

| **S3 Table. Multivariate linear regression analysis between gait speed and clinical and laboratory variables.** | | | | | | |
| --- | --- | --- | --- | --- | --- | --- |
|  | Model 1 | | Model 2 | | Model 3 | |
|  | β | P | β | P | β | P |
| Age | −0.10 | 0.053 | −0.11 | 0.052 | −0.12 | 0.027 |
| Body height | 0.07 | 0.22 | 0.06 | 0.24 | 0.05 | 0.360 |
| Body weight | −0.12 | 0.014 | −0.12 | 0.017 | −0.12 | 0.012 |
| Duration of RA disease | −0.03 | 0.58 | −0.03 | 0.61 | −0.01 | 0.92 |
| Steinbrocker Stage | −0.02 | 0.71 | −0.02 | 0.72 | −0.06 | 0.36 |
| DAS28-CRP | −0.14 | 0.0072 | −0.13 | 0.0096 |  |  |
| CDAI |  |  |  |  | −0.12 | 0.015 |
| CRP |  |  |  |  | 0.07 | 0.14 |
| RF positive |  |  | −0.04 | 0.45 | −0.06 | 0.24 |
| ACPA positive |  |  | 0.03 | 0.50 | 0.04 | 0.43 |
| Steroid use | −0.07 | 0.13 | −0.07 | 0.12 | −0.09 | 0.049 |
| Methotrexate use | 0.13 | 0.0055 | 0.12 | 0.0065 | 0.13 | 0.0033 |
| bDMARDs use | 0.04 | 0.42 | 0.03 | 0.47 | 0.04 | 0.44 |
| Interstitial lung disease | −0.15 | 0.0015 | −0.15 | 0.0015 | −0.16 | 0.0008 |
| Knee extension strength | 0.41 | <0.0001 | 0.41 | <0.0001 | 0.41 | <0.0001 |
| Total number of THA, TKA and TAA | −0.06 | 0.22 | −0.06 | 0.21 | −0.07 | 0.16 |
| β values represent standardized partial regression coefficient. R^2^ for model 1, model 2 and model 3 are 0.432, 0.433 and 0.434, respectively. P values calculated by ANOVA were < 0.0001 in all the three models. | | | | | | |
