# Supplementary material for: Factors affecting walking ability in female patients with rheumatoid arthritis
Source: PLoS One. 2018 Mar 27;13(3):e0195059. doi: 10.1371/journal.pone.0195059 (PMC5870996; doi:10.1371/journal.pone.0195059)
Supplement: S4 Table — Data are mean ± standard deviation or n (%). P value were derived from Wilcoxon rank-sum test or chi-square test. (DOCX) [file pone.0195059.s004.docx]

| **S4 Table. Differences between patients with methotrexate use and patients without methotrexate use** | | | |
| --- | --- | --- | --- |
|  | With methotrexate use | Without methotrexate use |  |
|  | (n=216, 67.9% ) | (n=102, 32.1%) | P |
| Step length, cm | 54.0 ± 11.0 | 50.1 ± 13.2 | 0.019 |
| Cadence, steps/min | 113.6 ± 11.3 | 109.3 ± 15.6 | 0.055 |
| Gait speed, m/s | 1.03 ± 0.26 | 0.93 ± 0.29 | 0.0031 |
| Age, years | 60.8 ± 12.5 | 63.6 ± 15.0 | 0.013 |
| Body height, cm | 156.1 ± 6.6 | 154.0 ± 7.8 | 0.028 |
| Body weight, kg | 51.6 ± 8.2 | 50.0 ± 8.7 | 0.057 |
| Body mass index, kg/m^2^ | 21.2 ± 3.3 | 21.1 ± 3.2 | 0.58 |
| Duration of RA disease, years | 13.1 ± 12.6 | 14.1 ± 12.2 | 0.20 |
| Steinbrocker Stage, no. | I; 41, II; 51, | I; 25, II; 23, | 0.70 |
|  | III; 38, IV; 86 | III; 18, IV; 36 |  |
| DAS28-CRP | 1.91 ± 0.81 | 2.04 ± 0.90 | 0.19 |
| CDAI | 5.70 ± 6.50 | 6.39 ± 5.77 | 0.083 |
| TJC, no. | 0.88 ± 1.47 | 0.86 ± 1.78 | 0.29 |
| SJC, no | 0.87 ± 1.41 | 0.75 ± 1.53 | 0.13 |
| dVAS | 10.1 ± 11.4 | 10.5 ± 12.9 | 0.95 |
| pVAS | 26.7 ± 23.8 | 36.3 ± 25.8 | 0.0007 |
| CRP, mg/dl | 0.29 ± 0.62 | 0.45 ± 0.74 | 0.15 |
| RF positive, no. (%) | 156 (72.2) | 77 (75.5) | 0.54 |
| ACPA positive, no. (%) | 172 (79.6) | 81 (79.4) | 0.20 |
| HAQ | 0.68 ± 0.70 | 0.98 ± 0.84 | 0.0021 |
| mHAQ | 0.36 ± 0.50 | 0.58 ± 0.67 | 0.024 |
| Steroid use, no. (%) | 62 (28.7) | 45 (44.1) | 0.0066 |
| bDMARDs use, no (%) | 86 (39.8) | 47 (46.1) | 0.29 |
| Interstitial lung disease, no. (%) | 28 (13.0) | 22 (21.6) | 0.049 |
| Knee extension strength, N | 1695.8 ± 648.1 | 1641.6 ± 782.6 | 0.20 |
| Total number of THA, TKA and TAA, no. | 0.30 ± 0.80 | 0.27 ± 0.73 | 0.97 |
| Data are mean ± standard deviation or n (%). P value were derived from Wilcoxon rank-sum test or chi-square test. | | | |
